# Chronic Unilateral Hearing Loss Disrupts Neural Tuning to Sound-Source Azimuth in the Rat Primary Auditory Cortex

**DOI:** 10.3389/fnins.2019.00477

**Published:** 2019-05-10

**Authors:** Xiuwen Wang, Jing Liu, Jiping Zhang

**Affiliations:** Key Laboratory of Brain Functional Genomics, Ministry of Education, NYU-ECNU Institute of Brain and Cognitive Science at NYU Shanghai, School of Life Sciences, East China Normal University, Shanghai, China

**Keywords:** hearing loss, auditory cortex, azimuth tuning, sound localization, plasticity

## Abstract

Accurate sound localization requires normal binaural input and precise auditory neuronal representation of sound spatial locations. Previous studies showed that unilateral hearing loss profoundly impaired the sound localization abilities. However, the underlying neural mechanism is not fully understood. Here, we investigated how chronic unilateral conductive hearing loss (UCHL) affected the neural tuning to sound source azimuth in the primary auditory cortex (AI). The UCHL was manipulated by the removal of tympanic membrane and malleus in the right ear of young (P14) rats and adult (P57) rats. We recorded the azimuth tuning of neurons in the left AI contralateral to the operated ear in the two groups of rats that experienced 2 months of UCHL, and in the left AI of age-matched control rats. We found that AI neurons in control rats showed predominant preference to sound from contralateral azimuths. However, UCHL weakened the cortical neuronal representation of contralateral azimuths on the operated ear side and strengthened the cortical neuronal representation of ipsilateral azimuths on the intact ear side. This effect was stronger in rats with UCHL at young age than in rats with UCHL in adulthood. Moreover, UCHL degraded the azimuth selectivity and azimuth sensitivity of AI neurons, and this effect was stronger in rats with UCHL in adulthood than in rats with UCHL at young age. These findings highlight a remarkable age-related experience-dependent plasticity of neural tuning to sound source azimuth in AI, and imply a neural mechanism for the impacts of chronic UCHL on sound localization abilities.

## Introduction

The ability to accurately localize and segregate different sound sources is a fundamental acoustical process for human and animals in analyzing signal sounds, monitoring acoustical environment, and guiding subsequent behavioral responses. To localize a sound source, the auditory system relies on the processing of sound spectral, temporal, and level information reaching to the two ears. Unilateral hearing impairments profoundly affect the sound localization ability of human subjects with hearing loss compared to normal hearing counterparts (Humes et al., 1980; Wilmington et al., 1994). Therefore, normal binaural hearing is necessary for accurate sound localization.

The neural mechanism for processing sound spatial information has been investigated in many previous studies. Behavioral studies by focal lesion or deactivation in the auditory cortex have shown that the AI is necessary for sound localization (Jenkins and Merzenich, 1984; King et al., 2007; Malhotra and Lomber, 2007), and that AI in each hemisphere mainly contributes to localizing sounds in the contralateral auditory space (Jenkins and Merzenich, 1984; Malhotra and Lomber, 2007). Studies in normal hearing animals have demonstrated that many neurons in AI were sensitive to sound source azimuth, i.e., they responded strongly to sound stimuli at some azimuths but poorly at others. Most of the neurons in AI preferred sound stimuli from the contralateral field, and only a small proportion of neurons preferred the sound stimuli from midline or ipsilateral field. This has been demonstrated in cats (Imig et al., 1990; Rajan et al., 1990; Barone et al., 1996; Eggermont and Mossop, 1998), rats (Yao et al., 2013; Gao et al., 2018), and monkeys (Woods et al., 2006). Studies also showed that many azimuth-sensitive neurons in the AI of adult cats determined in binaural conditions immediately became insensitive to sound source azimuth when determined with one ear plugged (Samson et al., 1994), demonstrating the importance of normal binaural input in the spatial sensitivity of AI neurons.

Abnormal binaural hearing is often found in patients with unilateral hearing loss, especially in very young children. During postnatal hearing development, a population of young children experience short-term or chronic UCHL associated with otitis media with effusion. The UCHL attenuates sound transmission to the inner ear with the cochlea remaining largely intact, and produces an imbalance in inputs between the two ears. The UCHL distorts the binaural information for localizing sound source, i.e., the interaural time differences and interaural level differences. Thus, understanding how the neurons in the auditory cortex respond to chronic UCHL at various postnatal ages can provide insights into the impacts of chronic otitis media with effusion and other disorders that affect sound transmission through the external or middle ear. In animal studies, UCHL can be induced by unilateral earplugging (Moore et al., 1989), monaural ear canal ligation (Popescu and Polley, 2010), and monaural disruption of tympanic membrane and ossicles (Tucci et al., 1999; Sumner et al., 2005). These manipulations have become important tools for studying the experience-dependent plasticity in the auditory system. Unilateral ear canal ligation in young rats caused distorted tonotopic maps and a reorganized aural dominance in AI. The representation of the ligated ear in AI was weakened and the representation of the ear with normal hearing was strengthened (Popescu and Polley, 2010). UCHL in young gerbils significantly decreases the metabolic activity in the major ascending projections of the manipulated ear in the central auditory system (Tucci et al., 1999). The most extreme condition of abnormal binaural input is unilateral deafness, e.g., congenital single-sided deafness, bilateral deafness equipped only with one cochlear implant, and unilateral cochlear ablation. The unilateral deafness completely eliminates the input from one of the two ears. Previous studies, by recording local field potentials from the cortical surface of cats, showed that single-sided deafness within an early sensitive period led to unilateral aural preference in favor to the hearing ear (Kral et al., 2013b), and that the plastic reorganizations exhibited hemispheric specificity (Kral et al., 2013a). Further studies by multi-unit recording demonstrated that unilateral hearing prevented non-specific reduction in cortical responsiveness in cat AI, but extensively reorganized aural dominance and binaural responses (Tillein et al., 2016). In addition, previous studies showed that postnatal unilateral cochlea ablation rewired the projection from the non-operated ear to the brainstem (Kitzes et al., 1995; Gabriele et al., 2000), disrupted the maintenance of the banded projections of the dorsal nucleus of the lateral lemniscus in the inferior colliculus ipsilateral to the ablation (Franklin et al., 2006), and led to a dramatic increase in the proportion of neurons in the inferior colliculus ipsilateral to the intact ear (McAlpine et al., 1997). These findings demonstrate a developmental and experience-dependent plasticity in the auditory system.

Accurate sound localization also relies on normal binaural hearing and precise processing of auditory spatial information in the auditory system during postnatal hearing development. In human subjects with congenital UCHL, the performance of sound localization was still below the control level after corrected congenital UCHL (Wilmington et al., 1994). It is possible that abnormal binaural input profoundly affects the neural representation of auditory space in the central auditory system and consequently impairs sound localization performance. However, at present we still have a poor understanding of how chronic UCHL at various postnatal ages affects the neural representation of auditory space in the auditory cortex. In the present study, we investigated whether identical UCHL manipulation carried out in young rats and adult rats produces the same outcome in the neural representation of spatial azimuth in the rat AI. Because previous studies have demonstrated a dominant preference of AI neurons to stimuli from the contralateral auditory space (Imig et al., 1990; Rajan et al., 1990; Barone et al., 1996; Eggermont and Mossop, 1998; Woods et al., 2006; Gao et al., 2018; Yao and Sanes, 2018), we here focused on studying the impacts of postnatal UCHL on the neural tuning to sound source azimuth in the AI contralateral the operated ear. We found that chronic UCHL carried out at both young and adult ages altered the azimuth preference, degraded the azimuth selectivity and sensitivity of AI neurons. Importantly, we found age-related impacts of UCHL on the azimuth tuning of AI neurons exhibited by azimuth preference as well as the azimuth sensitivity and azimuth selectivity of AI neurons.

## Materials and Methods

### Animals and Animal Groups

The Sprague Dawley rats of both sexes were obtained from an in-housing breed stock that originated from the breeding pairs purchased from Shanghai Jie Shi Jie Laboratory Animal Co., Ltd. (Shanghai, China). Four groups of rats were used: (1) Young unilateral conductive hearing loss group (YUCHL group): the rats were operated with UCHL surgery by removing the tympanic membrane and malleus in the right ear at postnatal (P) day 14 (P14), and were reared till the age of P74–P87. (2) Young control group (YCon group): the rats with no UCHL surgery at young age were reared till the age of P74–P87. (3) Adult unilateral conductive hearing loss group (AUCHL group): the rats were operated with UCHL surgery in the right ear at P57, and were reared till the age of P117–P130. (4) Adult control group (ACon group): rats with no UCHL surgery were reared till the age of P117–P130. All rats had free access to food and water, and were reared in the housing environments on 12 h light-dark cycles. The housing temperature was maintained at 20–24°C. The age of rats used for neurophysiological recording was P74–P87 for both the YUCHL group and the YCon group, and was P117–P130 for both the AUCHL group and the ACon group. The purpose of selecting rats within these age ranges for neurophysiological recording was to ensure that all rats used for data recording were in adulthood, and that the impacts of chronic UCHL on azimuth tuning of AI neurons in both the YUCHL group and the AUCHL group were determined within 2 weeks after the rats had 2 months of UCHL experiences.

In the present study, the neurophysiological data were obtained from a total of 95 rats including 29 rats in the YCon group, 27 rats in the YUCHL group, 20 rats in the ACon group, and 19 rats in the AUCHL group, respectively.

### Surgery Procedure for UCHL

Unilateral conductive hearing loss was induced in the right ear using the procedures similar to those described in previous studies (Tucci et al., 1999; Xu et al., 2007). Surgical anesthesia was induced with pentobarbital sodium (40 mg/kg i.p.) and was confirmed by a complete elimination of responses to nociceptive stimuli. In the right ear, both the tympanic membrane and the malleus were removed with jeweler’s forceps under microscope. Following the surgery the animals were allowed to fully recover under supervision and then were returned to their cages.

### Auditory Brainstem Response Measurement

Before doing single-unit recording in the auditory cortex, we measured the ABR from a subset of rats in each group. The main purpose of the ABR measurement was to evaluate the differences in hearing threshold between the intact left ear and the operated right ear with UCHL surgery in the two groups of rats with UCHL.

Rats were anesthetized with urethane (1.5 g/kg i.p.) and then placed in a stereotaxic frame in a double-walled sound-proof room. The inside walls and ceiling of the sound-proof room were covered by sound-absorbing foams to reduce acoustic reflections. The ABR measurements were obtained in a neurophysiology workstation (TDT3, Tucker-Davis Technologies, United States). The multifunction processor (RX6-A5), speaker amplifier (SA1), and multi-field magnetic speakers (MF1) were used to present closed-field acoustic stimuli. The output of the magnetic speakers was calibrated in a closed-field system from 2.0 to 48.0 kHz (sampling rate, 100 kHz) using a one-quarter inch condenser microphone (model 7016, ACO Pacific Inc.). The acoustic signal was present from the calibrated magnetic speaker coupled to the ear. The subdermal needle electrodes (Rochester Electro-Medical, Inc., United States), headstage (RA4LI), preamplifier (RA4PA), and RA16 medusa base station were used to record ABR signals. The electrodes were placed subcutaneously at the vertex (active), the mastoid ipsilateral to the acoustic signal source (reference), and the tail of the rats (ground). The ABR thresholds were measured independently for both ears with tone bursts (5 ms duration, 0.5 ms cosine ramps, 21 Hz repetition rate). The tone bursts were 4–36 kHz in frequency with 4 kHz increments, and 15 dB SPL to 85 dB SPL in level with 5 dB increments. The ABR signals were bandpass filtered (0.3–3 kHz), averaged from 512 stimulus pairs, and analyzed in BioSigRP software. The ABR Wave I threshold was defined as the lowest sound level that could reliably produce an acoustic stimulus-evoked peak which followed the progressive trend for decreasing amplitude and increasing latency obtained over the range of tested sound levels (Popescu and Polley, 2010). We determined the ABR Wave I threshold by visual inspections of ABR data, and by using a statistical measure, i.e., the lowest sound level that evoked a wave I with the peak-to-peak amplitude greater than two standard deviations of the background activity.

**FIGURE 1:**
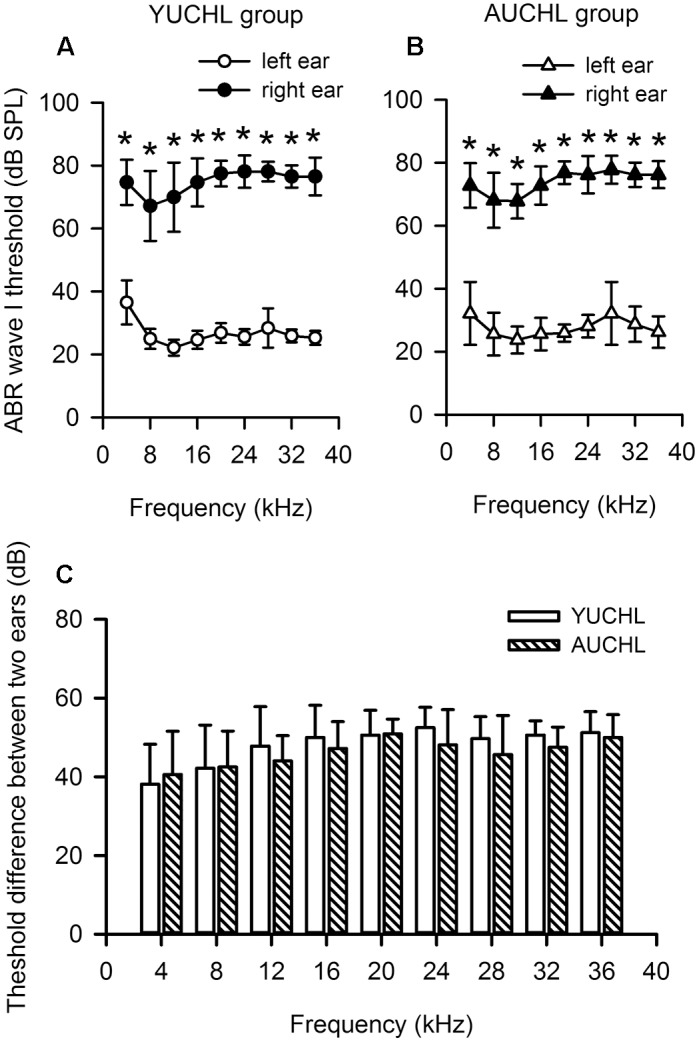
Unilateral conductive hearing loss (UCHL) elevated the auditory brainstem response (ABR) wave I threshold in the operated left ear. YUCHL group: young unilateral conductive hearing loss group; AUCHL group: adult unilateral conductive hearing loss group. Data (shown in mean and standard deviations) were from a subset of rats in the YUCHL group (*n* = 15) and the AUCHL group (*n* = 15). **(A,B)** The ABR wave I thresholds determined independently from the intact left ear and right ear with UCHL in both the YUCHL group **(A)** and the AUCHL group **(B)**. ^∗^ indicates significantly different in ABR wave I threshold compared to the corresponding data from the left ear (Wilcoxon Signed Rank Test, *p* < 0.001). Note that the ABR thresholds determined in the right ear with UCHL were higher than those in the intact left ear. **(C)** The average differences in ABR wave I thresholds (right ear threshold minus left ear threshold) determined at each frequency. At each frequency, the threshold differences were not significantly different between the two groups (Mann–Whitney test, all *p* > 0.05). See texts in the “Materials and Methods” section for details.

The ABR data demonstrated that UCHL surgery induced a significant hearing loss, and the average differences in hearing threshold between the intact ear and the operated ear were within the range of 38–53 dB across the frequencies tested (Figure 1A,B, Wilcoxon Signed Rank Test under each tested frequency condition, right ear vs. left ear, all *p* < 0.001 in both YUCHL group and AUCHL group). The average differences in hearing threshold between the intact left ear and the operated right ear did not show significant group differences between the YUCHL group and the AUCHL group (Figure 1C, Mann–Whitney test, YUCHL vs. and AUCHL, all *p* > 0.05 under each of the tested frequency condition). The effect of UCHL surgery on the hearing threshold of rats determined in the present study is comparable to that shown in previous studies in gerbils (Tucci et al., 1999).

### Animal Surgery for Single-Unit Recording in AI

Surgical anesthesia was induced with urethane (1.5 g/kg i.p.) and maintained by intraperitoneal infusion of urethane via an automatic microinfusion pump (WZ-50C6, China) during the surgery and neurophysiological recording. Atropine sulfate (0.01 mg/kg, sc) was given to reduce bronchial secretions. Body temperature was continuously monitored by a thermal probe and maintained at 37.5°C using a thermostatically controlled heating blanket. The surgical procedures were similar to that described in our previous studies (Jiang et al., 2015; Lu et al., 2016). Briefly, after the trachea of rats was cannulated, the dorsal and temporal skull were exposed. The temporal bone was exposed by partially removing the temporal muscle. A nail (4 cm long) was attached to the dorsal surface of the skulls with 502 super glue and dental cement. The head of the rat was then fixed by the nail to a head holder attached to a stainless steel platform. A craniotomy was performed over the left auditory cortex (contralateral to operated right ear in YUCHL rats and AUCHL rats). The dura was then removed, and the exposed cortex was kept moist by applying warm saline to the cortex.

### Systems for Acoustic Stimulus Presentation and Neurophysiological Recording in AI

Acoustic stimulus presentation and single-unit recording were performed through a neurophysiology workstation using TDT System 3 hardware and software (Tucker-Davis Technologies, United States) controlled by a PC. The portion of the workstation for acoustic stimulus presentation includes a multifunction processor (RX6-A5), an electrostatic loudspeaker driver (ED1), and a free field electrostatic loudspeaker (ES1). The acoustic stimuli were delivered through a free-field system. The distance between the loudspeaker and the center of the interaural axis of the rats was constant (20 cm) for all spatial positions tested in the present study. The loudspeaker was able to move freely in the frontal auditory space of the rats by a custom-made motor system. The spatial positions (i.e., azimuths and elevations) of the loudspeaker were monitored by two video cameras. The loudspeaker location directly in front of the rat was assigned to 0° in azimuth, and the loudspeaker elevation aligned to the center of the rat’s head was assigned 0° in elevation. In the present study, the loudspeaker elevation was fixed to 0° whereas the loudspeaker azimuth was varied from the left 90° (assigned to -90°) to right 90° (assigned to +90°) in the rat frontal auditory field by a remote control system outside the sound-proof room. The positive azimuths refer to the azimuths contralateral to the cortex (left cortex) being recorded, and the negative azimuths represent the azimuths ipsilateral to the cortex being recorded. The output of the loudspeaker was calibrated from 2.0 to 48.0 kHz (sampling rate, 100 kHz) using a one-quarter inch condenser microphone (model 7016, ACO Pacific Inc.) placed at the center of the interaural axis of rats and faced directly to the loudspeaker. The calibration data were stored in the computer for use in obtaining the desired sound pressure levels in decibel (dB SPLs, re: 20 μPa) within the calibrated frequency range. The acoustic stimuli were tonal stimuli with 50 ms in duration including 2.5 ms rise time and 2.5 ms fall time. The acoustic stimulus frequencies used in the present study did not exceed 46.0 kHz. The interstimulus interval was 1,000 ms.

The recording of neural responses in the auditory cortex to acoustic stimuli was conducted in the sound-proof room. Glass electrodes (1.0–2.0 MΩ impedance, filled with 2M NaCl) were advanced orthogonally to the pial surface of AI by a microdrive (SM-21, Narishige, Japan) that was remotely controlled outside the sound-proof room. Action potentials were recorded, amplified (×1,000), and band pass filtered (0.3–3.0 kHz) by a DAM80 amplifier (WPI, United States), and then fed into a pre-amplifier RA8GA, a RA16 Medusa base station (TDT), and PC for online and offline data processing. The signal was also sent to a digital oscilloscope (TDS 2024, United States) for display and an audio monitor for listening.

### Data Collection and Acoustic Stimulus Presentation Paradigm

At the beginning of single-unit recording in the left auditory cortex, the free-field loudspeaker was placed at 30° in azimuth and 0° in elevation in the rat frontal auditory space. The procedure to locate the AI of rats was similar to that described in our previous study (Lu et al., 2016). Preliminary electrode penetrations were made perpendicular to the pial surface of the auditory cortex of each rat to construct a coarse tonotopic map. The positions of the penetrations were within the range of 3.0–5.5 mm posterior to Bregma in anterior–posterior direction, and 3.0–5.0 mm to the Bregma in dorsal–ventral direction. The depth of the penetrations was limited to the range of 300–800 μm below the pial surface. The AI was defined based on electrophysiological properties such as short-latency responses to acoustic stimuli and increasing characteristic frequencies (CFs) of neurons in a caudal to rostral direction (Doron et al., 2002). Once a single unit was well isolated (based on the constancy of amplitude and waveform of evoked action potentials) in AI, the CF of the neuron was determined. We first audio-visually determined the frequency-level ranges that the neuron responded at both 30° and -30° in azimuth. At one of two azimuths which the neuron responded to tonal stimuli at the lowest sound level, we determined the CF of the neuron using a stimulus matrix varied with sound levels (0 to 80 dB SPL in 10-dB steps) and sound frequencies (frequency range: previously audio-visually determined, interval: 1.0 kHz). The CF was the frequency which evoked a response of the neuron at the lowest threshold level. We then determined the rate-level function at the CF and within the level range of 0–80 dB SPL with 10-dB steps. Each stimulus was repeated 20 times. Due to low spontaneous activities in anesthetized rat AI, the MT of the neuron was arbitrarily defined as the sound level that elicited 20% of the maximal spike count in the rate-level function. We used this sound level as the reference level to measure the azimuth tuning curves (or azimuth functions) of the neuron. The azimuth functions of the neuron were determined at the CF, and with an azimuth-level stimulus matrix varied with azimuths (-90°, -70°, -50°, -30°, -10°, 0°, +10°, +30°, +50°, +70°, and +90°) and sound levels (MT+5 dB, MT+10 dB, and MT+20 dB). If the neuron was still available for recording after the three azimuth functions were determined, an additional rate-level function of the studied neuron was obtained to evaluate the stability of the neuron’s response. If the neural responses to the same stimuli varied greater than 20%, the data were excluded from further analysis.

**FIGURE 2:**
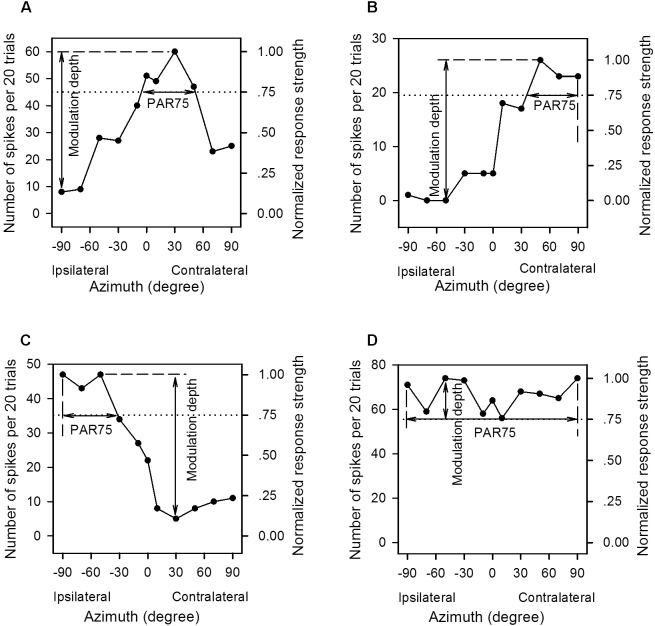
The method to determine the preferred azimuth range and the modulation depth from the azimuth functions of the primary auditory cortex (AI) neurons. **(A–D)** For each panel, the left *y*-axis indicates the number of spikes evoked by per 20 trials of tonal stimuli, and the right *y*-axis indicates the normalized response strength. The spike counts are normalized to the response strengths relative to the maximum spike count in each azimuth function, and the maximum response strength is 1.00. The *x*-axis indicates the speaker location in degree contralateral (positive azimuth) or ipsilateral (negative azimuth) to the cortex (left cortex) being recorded, and 0° is midline azimuth. The dotted line indicates 0.75 in normalized response strength. The range of the horizontal solid line with double arrowhead in each panel is defined as the PAR75, i.e., the preferred azimuth range over which the normalized response strengths are ≥0.75. The range of the vertical solid line with double arrowhead is defined as the modulation depth, i.e., the difference value between the maximum and the minimum normalized response strength in each azimuth function. See texts in the “Materials and Methods” section for details.

The AI neurons tested in the present study all responded transiently to the onset of the sound stimuli. The neural responses to the azimuth-level stimulus matrix were determined based on the total number of spikes per 20 trials of sound stimuli evoked within the sound duration. For each neuron, we plotted the azimuth function at each sound level in both spike counts and normalized response strength (Figure 2). We normalized the responses in spike counts by calculating the response strength relative to the maximum spike count in each azimuth function, and the maximum response strength was 1.0 (Figure 2, the left *y*-axis indicates spike counts evoked by 20 trials of stimuli, and the right *y*-axis indicates the normalized response strength). For each azimuth function, we determined the preferred azimuth range over which the normalized response strength was ≥0.75 (PAR75). The location of the PAR75 in the horizontal plane was used as a measure of azimuth preference whereas the width of the PAR75 was used as a measure of azimuth selectivity. In each panel of Figure 2, the dotted horizontal line indicates the normalized response strength at 0.75, and the horizontal double-headed arrow show the PAR75 determined for each azimuth function. If the dotted line intersects with both sides of the azimuth function, the PAR75 was the azimuth range between the two intersects (Figure 2A, horizontal line with double arrowhead). For the unbounded azimuth function, if the dotted line intersects with only one side of the azimuth function, the PAR75 was defined as the azimuth range from the intersect of the dotted line with the azimuth function to the contralateral azimuth limit (i.e., azimuth +90°, Figure 2B) or the ipsilateral azimuth limit (i.e., azimuth -90°, Figure 2C). If the dotted line did not intersect with either side of the azimuth function, the PAR75 was defined as the azimuth range from -90° to +90° (i.e., PAR75 = 180°, Figure 2D). It should be noted that the method to determine PAR75 in the unbounded azimuth functions (Figure 2B–D) underestimates the actual extent of PAR75. If an azimuth function has multiple PAR75s, the PAR75 width of the neuron was defined as the sum of the extent of these PAR75s. In addition, the modulation depth of each azimuth function was defined as the difference in normalized response strength between the maximum value and the minimum value (Figure 2A–D, vertical lines with double arrowhead). The modulation depth of the azimuth function was used as a measure of azimuth sensitivity.

Statistical analyses were performed in SPSS 13.0, and a criterion of *p* < 0.05 was considered as significantly different between groups.

## Results

For each neuron that was available for recording in the rat AI, we determined the azimuth functions at its CF and at the sound levels of MT+5 dB, MT+10 dB, and MT+20 dB, respectively. The data of the azimuth functions from 278 neurons were obtained in the AI of the four groups of rats, including 78 neurons in the YCon group, 73 neurons in the YUCHL group, 61 neurons in the ACon group, and 66 neurons in the AUCHL group. The CF ranges of these AI neurons were 4.0–44.0 kHz in the YCon group, 5.0–41.0 kHz in the YUCHL group, 8.0–41.0 kHz in the ACon group, and 4.2–35.0 kHz in the AUCHL group.

Unilateral conductive hearing loss induced an increase in the MTs of AI neurons contralateral to operated ear in comparison with the MTs of AI neurons in the age-matched control rats. The MTs of AI neurons were significantly higher in the YUCHL group than in the YCon group (YUCHL vs. YCon in mean and standard error, 45.00 ± 1.85 dB SPL vs. 31.46 ± 1.11 dB SPL, Mann–Whitney Test, *z* = -5.273, *p* < 0.001). Similarly, the MTs of AI neurons were significantly higher in the AUCHL group than in the ACon group (AUCHL vs. ACon in mean and standard error, 54.16 ± 2.27 dB SPL vs. 30.77 ± 1.28 dB SPL, Mann–Whitney Test, *z* = -5.273, *p* < 0.001). In the two UCHL groups, the MTs of AI neurons were significantly higher in the AUCHL group than in the YUCHL group (Mann–Whitney Test, *z* = -2.942, *p* = 0.003). No significant differences were found in MTs between the two control groups (YCon vs. ACon, Mann–Whitney Test, *z* = -0.577, *p* = 0.564).

### Classifications of the Azimuth Functions of Rat AI Neurons

**FIGURE 3:**
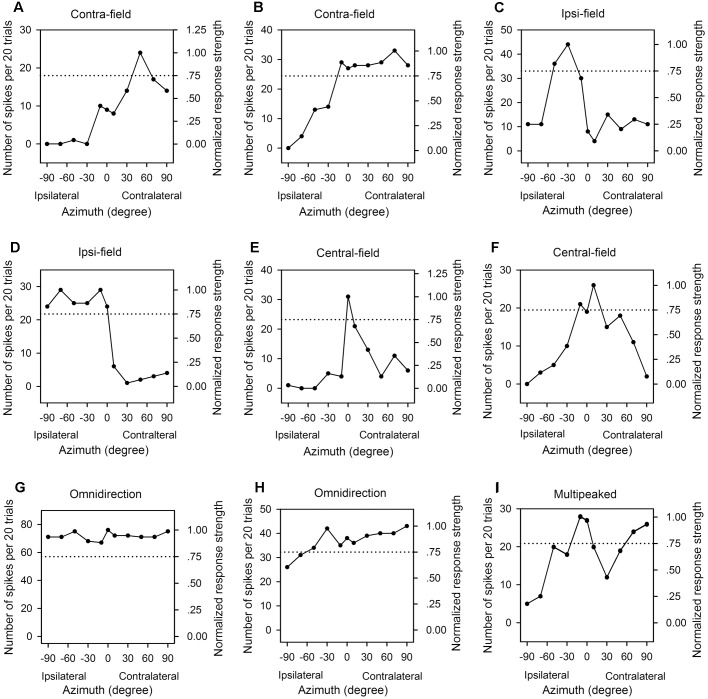
The azimuth functions of nine representative AI neuron with various azimuth preferences. Each panel shows the data from one neuron, the left *y*-axis indicates the number of spikes per 20 trials, and the right *y*-axis indicates the normalized response strength. The *x*-axis indicates the loudspeaker locations in azimuth (degree). The dotted line in each panel indicate 0.75 in normalized response strength. **(A,B)** Contralateral azimuth preference (Contra-field). **(C,D)** Ipsilateral azimuth preference (Ipsi-field). **(E,F)** Central azimuth preference (Central-field). **(G,H)** Omnidirection. **(I)** Multipeak. The frequencies (kHz) and the levels (dB SPL) of sounds used to determine the azimuth functions are as follows: **(A)** 8.0 kHz, 45 dB SPL; **(B)** 9.0 kHz, 60 dB SPL; **(C)** 11.0 kHz, 45 dB SPL; **(D)** 34.0 kHz, 35 dB SPL; **(E)** 14.0 kHz, 30 dB SPL; **(F)** 10.1 kHz, 40 dB SPL, respectively.

In the frontal auditory space, the azimuths were categorized into contralateral azimuths (from +10° to +90°, not including +10°), central azimuths (from -10° to +10°, including both -10° and +10°), and ipsilateral azimuths (from -10° to -90°, not including -10°) relative to the cortex being recorded. We determined the PAR75 from each azimuth function of an AI neuron measured at a specified sound level, and then categorized the azimuth function at that sound level based on the location and the extent of PAR75. For each azimuth function, it was classified as “contra-field preference” if the PAR75 was restrictively or predominantly distributed within the range of contralateral azimuths (Figure 3A,B). The azimuth function was assigned to “ipsi-field preference” if the PAR75 was restrictively or predominantly distributed within the range of ipsilateral azimuths (Figure 3C,D). The azimuth function was considered as “central-field preference” if the PRA75 was restrictively or predominantly distributed within the range of central azimuths and with a decline of at least 50% in response on both sides of the maximum response (Figure 3E,F). The azimuth function was classified as “omnidirection” if the responses determined at all of the tested azimuths were ≥75% of the maximum response (Figure 3G), or if the responses determined at ≥9 of the 11 tested azimuths were ≥75% of the maximum response in the function and the responses at all tested azimuth were ≥50% of the maximum response (Figure 3H). The azimuth function was assigned to “multipeak” if there were two or more widely separated peaks in the function, and the PAR75s were distributed within at least two of the three ranges in spatial azimuth, i.e., contralateral azimuths, central azimuths, and ipsilateral azimuths, respectively (Figure 3I). If the azimuth function exhibited two peaks and the PAR75s were located within the same lateral-field, the azimuth function was categorized as either contra-field preference or ipsi-field preference.

**FIGURE 4:**
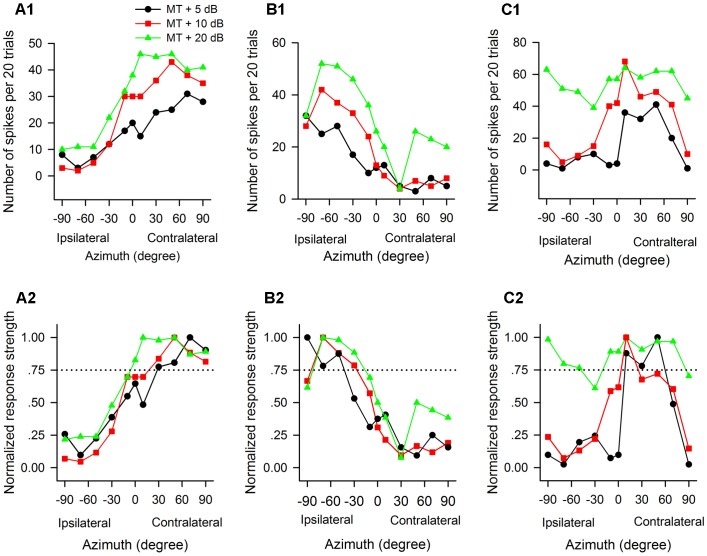
The azimuth functions of three representative AI neurons vary with the change of sound stimulus levels. The data in each column are from one neuron. **(A1–C1)** Azimuth functions shown in number of spikes per 20 trials. **(A2–C2)** Azimuth functions shown in normalized response strength. MT, minimum threshold of the neuron. Curves with different symbols are the azimuth functions determined at the sound levels of MT+5 dB, MT+10 dB, and MT+20 dB, respectively (see the legend in **A1**). The CF (kHz) and MT (dB SPL) of the three neurons are as follows: **(A1,A2)** 13.0 kHz, 35 dB SPL; **(B1,B2)** 34.1 kHz, 30 dB SPL; **(C1,C2)** 35.0 kHz, 20 dB SPL, respectively. Note that the azimuth functions and the azimuth preferences of the three neurons varied with the change of sound levels.

The azimuth functions of AI neurons varied with the tested sound levels. Figure 4 shows the azimuth functions of three AI neurons determined at 5 dB, 10 dB, and 20 dB above the MT of each neuron. It’s evident that the azimuth functions varied with neurons, and varied with sound levels for each neuron. The neuron A preferred sound stimuli from contralateral azimuths at MT+5 dB and MT+10 dB conditions, and preferred sound stimuli predominately from contralateral azimuths at MT+20 dB condition (Figure 4A1,A2). The preferred azimuth ranges of neuron A increased with increasing sound levels. Neuron B showed clear preference to sound stimuli from ipsilateral azimuths, and the preferred azimuth range of this neuron also varied with sound levels (Figure 4B1,B2). Neuron C showed preference to sound stimuli from contralateral azimuths at MT+5 dB, and preference to central azimuths at MT+10 dB (Figure 4C1,C2); in contrast, neuron C showed no preference to sound azimuths (i.e., omnidirection) at MT+20 dB condition (Figure 4C1,C2).

### The Effects of UCHL at Different Postnatal Ages on the Azimuth Functions of AI Neurons

**FIGURE 5:**
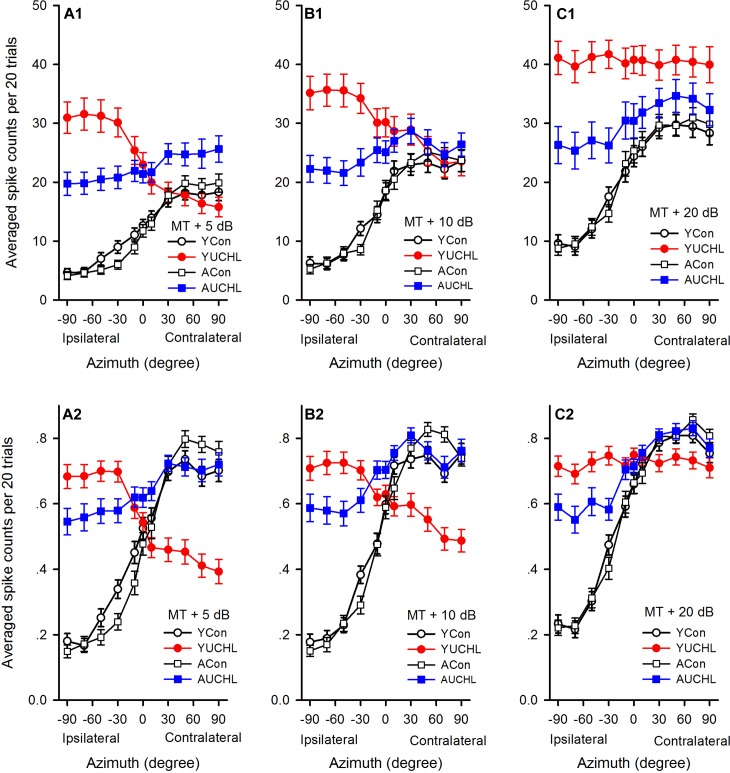
Comparison of the average azimuth functions shown in spikes counts and normalized response strength for the population of AI neurons in the four groups of rats. **(A1–C1)** The average azimuth functions shown in spike counts. **(A2–C2)** The average azimuth functions shown in normalized response strength. The sound levels used to determine the azimuth functions are shown in the legend of each panel. YCon, young control; YUCHL, young unilateral conductive hearing loss; ACon, adult control; AUCHL, adult unilateral conductive hearing loss. The data are shown in mean and standard error.

For the population of AI neurons in each group of rats, we determined the average azimuth functions by calculating the means and standard errors of spike counts at each azimuth under various acoustic stimulus conditions (Figure 5A1–C1). We then did data analysis (Repeated measure ANOVA with LSD *post hoc* tests) to determine the azimuth preferences of the average azimuth functions shown in spike counts for each group. The data demonstrated a clear preference to sound stimuli from contralateral azimuths for neurons in both the YCon group and the ACon group. For each of the average azimuth functions in the YCon group, the average spike counts at each of the contralateral azimuths (30°, 50°, 70°, and 90°) were greater than those at each of the ipsilateral azimuths (-30°, -50°, -70°, and -90°) (all *p* < 0.001) and at each of the central azimuths (-10°, 0°, and 10°) (all *p* ≤ 0.01); similarly, for each average azimuth function in the ACon group, the average spike counts at each of the contralateral azimuths (30°, 50°, 70°, and 90°) were greater than those at each of the ipsilateral azimuths (-30°, -50°, -70°, and -90°) (all *p* < 0.001) and at each of the central azimuths (-10°, 0°, and 10°) (all *p* < 0.001). In contrast, the average azimuth functions in the YUCHL group exhibited ipsilateral azimuth preference under the conditions of MT+5 dB and MT+10 dB (Figure 5A1,B1) but showed no azimuth preference under MT+20 dB condition (Figure 5C1). Under the conditions of MT+5 dB and MT+10 dB, for each of the average azimuth functions in the YUCHL group, the average spike counts determined at each of the ipsilateral azimuths (-30°, -50°, -70°, and -90°) were greater than those determined at each of the contralateral azimuths (30°, 50°, 70°, and 90°) (all *p* < 0.05); however, no significant differences were found in average spike counts determined at any two tested azimuth pairs in the average azimuth function under MT+20 dB condition (all *p* > 0.05). For the average azimuth functions in the AUCHL group, they exhibited weak preference to the contralateral azimuths: under MT+20 dB condition, the average spikes counts determined at each of the contralateral azimuths (30°, 50°, 70°, and 90°) were greater than those at each of the ipsilateral azimuths (-30°, -50°, -70°, and -90°) (all *p* < 0.05); however, under both MT+5 dB and MT+10 dB conditions, the average spikes counts determined at only a portion of the contralateral azimuths were greater than those at a portion of the ipsilateral azimuths.

We then compared the average azimuth functions (Figure 5A1–C1) for population of neurons among the four groups (one-way ANOVA with LSD *post hoc* tests). We found that the average spike counts were not significantly different between the YCon group and the ACon group at each of the azimuths tested (Figure 5A1–C1; YCon vs. ACon, all *p* > 0.05). In comparison with the data in both the YCon group and the ACon group, the data in both the YUCHL group and the AUCHL group demonstrated that UCHL consistently elevated the responses to stimuli from each of the ipsilateral azimuths (the intact ear side, -30°, -50°, -70°, and -90°) (Figure 5A1–C1; at MT+5 dB, all *p* < 0.001, at MT+10 dB, all *p* < 0.001, at MT+20 dB, all *p* < 0.01). Moreover, this elevation of responses to stimuli from the ipsilateral azimuths was more evident in the YUCHL group than in the AUCHL group (Figure 5A1–C1; YUCHL vs. AUCHL at each of the ipsilateral azimuths, all *p* < 0.001). For the responses to stimuli from each of the contralateral azimuths (30°, 50°, 70°, and 90°), under MT+5 dB condition, the average spike counts were greater in the AUCHL group than in any one of the other three groups (Figure 5A1, all *p* < 0.05) whereas the average spike counts in YUCHL group were not significantly different from the two control groups (Figure 5A1, all *p* > 0.05); under MT+10 dB condition, we did not find significant differences in spike counts at each of the contralateral azimuths among the four groups (Figure 5B1, all *p* > 0.05); under MT+20 dB condition, at each of the contralateral azimuths, the average spike counts were greater in the YUCHL group than in the two control groups (Figure 5C1, all *p* < 0.01) whereas the average spike counts in the AUCHL group were not significantly different from the two control groups (Figure 5C1, all *p* > 0.05). In addition, we only found significant differences in the average spike counts between the YUCHL group and the AUCHL group at the azimuth 90° (Figure 5C1, YUCHL vs. AUCHL, at the azimuth 90°, *p* = 0.04; at the azimuths 30°, 50°, and 70°, all *p* > 0.05). Therefore, the UCHL at different postnatal periods induced significantly different impacts on the azimuth tuning of AI neurons.

Because the spike counts in the azimuth functions varied with neurons, we compared the mean normalized azimuth functions among the four groups. For each azimuth function, we normalized the spike counts into the response strength relative to the maximum spike count in the azimuth function, and the maximum response strength was 1.0 (Figure 2–4). At each of the three sound levels tested (i.e., MT+5 dB, MT+10 dB, and MT+20 dB, respectively), we averaged the normalized response strength in the azimuth functions for the population of AI neurons in each group of rats, and the data were plotted into the mean normalized azimuth functions (Figure 5A2–C2). The population data demonstrated that the mean normalized azimuth functions in both the YCon group and the ACon group exhibited clear preferences to sound stimuli from contralateral azimuths (Figure 5A2–C2). Statistical analysis (repeated measure ANOVA with LSD *post hoc* tests) showed that, for each normalized mean azimuth function in the YCon group, the average response strength at each of the contralateral azimuths (30°, 50°, 70°, and 90°) was greater than those at each of the central azimuths (-10°, 0°, and 10°; all *p* < 0.001), and was also greater than those at each of the ipsilateral azimuths (-30°, -50°, -70°, and -90°; all *p* < 0.001). Similar findings were shown in the mean azimuth functions in the ACon group. In contrast, the mean normalized azimuth functions for YUCHL group exhibited ipsilateral azimuth preference under the conditions of MT+5 dB and MT+10 dB (Figure 5A2,B2), but showed no azimuth preference at MT+20 dB (Figure 5C2). Under the conditions of MT+5 dB and MT+10 dB, for each of the mean normalized azimuth functions in the YUCHL group, the average response strength determined at each of the ipsilateral azimuths (-30°, -50°, -70°, and -90°) was greater than those determined at each of the contralateral azimuths (30°, 50°, 70°, and 90°) (all *p* < 0.05); however, no significant differences were found in the average response strengths determined at any two tested azimuth pairs under MT+20 dB condition (all *p* > 0.05). For the AUCHL group, the mean azimuth functions of AI neurons exhibited weak preference to contralateral azimuths. For each of the three mean azimuth functions, the mean response strength determined at each of the contralateral azimuths (30°, 50°, 70°, and 90°) was greater than those determined at each of the ipsilateral azimuths (-30°, -50°, -70°, and -90°) (all *p* < 0.05).

In each of the mean normalized azimuth functions in Figure 5, the difference value between the maximum and the minimum average response strength reflect the azimuth sensitivity for population AI neurons. The larger difference value indicates greater azimuth sensitivity. The difference values in each of the mean normalized azimuth functions in the four groups were as follows: at MT+5 dB, 0.57 in YCon group, 0.31 in YUCHL group, 0.65 in ACon group, and 0.19 in AUCHL group (Figure 5A2); at MT+10 dB, 0.58 in YCon group, 0.23 in YUCHL group, 0.68 in ACon group, and 0.24 in AUCHL group (Figure 5B2); at MT+20 dB, 0.59 in YCon group, 0.04 in AUCHL group, 0.64 in ACon group, and 0.27 in AUCHL group (Figure 5C2). These data indicate that the difference values in both the YCon group and the ACon group were larger than those in both the YUCHL group and the AUCHL group. These data suggest that UCHL induced a general decrease of azimuth sensitivity of neurons in the AI contralateral to the operated ear.

### The Effect of UCHL at Different Postnatal Ages on the Azimuth Preferences of AI Neurons

**FIGURE 6:**
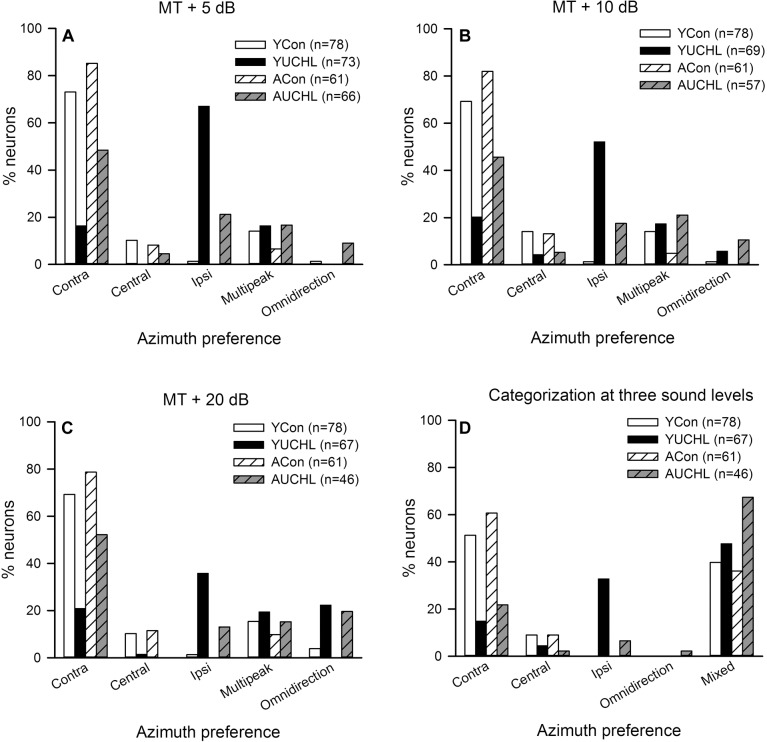
Comparison of the azimuth preference of AI neurons in the four groups of rats. Contra, contra-field preference; Central, central-field preference; Ipsi, ipsi-field preference. Panels **(A–C)** show the percentage of AI neurons with different azimuth preferences determined at each of the three sound levels, i.e., MT+5 dB, MT+10 dB, and MT+20 dB, respectively. Panel **(D)** shows the comparison of the overall azimuth preferences of AI neurons categorized from the azimuth functions determined at all the three sound levels in the four groups. The azimuth preference of an AI neuron was categorized as “mixed” when the azimuth preferences determined at the three sound levels are inconsistent. The ‘n’ in the legend of each panel show the number of neurons included in the data analysis.

For each AI neuron, we determined the PAR75 from each of the azimuth functions determined under the tested sound levels. As shown in Figure 2, based on the location of PAR75 in the horizontal plane, we assigned the azimuth preference of the neuron under each sound level condition into one of the five categories: contra-field preference, ipsi-field preference, central-field preference, omnidirection, and multipeak. Figure 6 shows the percentage of AI neurons in each category of azimuth preference in the four groups of rats. It’s evident that, at the tested sound levels, majority of the neurons in both YCon group and ACon group exhibited contra-field preference whereas a small proportion of neurons showed other types of azimuth preference. However, UCHL decreased the percentage of neurons with contra-field preference and increased the percentage of neurons with ipsi-field preference in both YUCHL group and AUCHL group, and this tendency is more evident in the YUCHL group than in the AUCHL group (Figure 6A–C). The data for the percentages of AI neurons with contra-field preference in the four groups were as follows: at MT+5 dB, YCon vs. YUCHL, 73.08% vs. 16.44%; ACon vs. AUCHL, 85.25% vs. 48.49%. At MT+10 dB, YCon vs. YUCHL, 69.23% vs. 20.29%; ACon vs. AUCHL, 81.97% vs. 45.61%. At MT+20 dB, YCon vs. YUCHL, 69.23% vs. 20.90%; ACon vs. AUCHL, 78.69% vs. 52.17%. In addition, the data for the percentages of neurons with ipsi-field preference in the four groups were as follows: at MT+5 dB, YCon vs. YUCHL, 1.28% vs. 67.12%; ACon vs. AUCHL, 0.00% vs. 21.21%. At MT+10 dB, YCon vs. YUCHL, 1.28% vs. 52.17%; ACon vs. AUCHL, 0.00% vs. 17.54%. At MT+20 dB, YCon vs. YUCHL, 1.28% vs. 35.82%; ACon vs. AUCHL, 0.00% vs. 13.04%. Moreover, UCHL increased the proportion of omnidirection neurons in both YUCHL group and AUCHL group at MT+10 dB and MT+20 dB conditions, indicating a loss of azimuth selectivity of some AI neurons (Figure 6B,C).

We then classified the azimuth preference of each AI neuron based on the overall azimuth preferences tested at the three sound levels (Figure 6D). If the azimuth preference of an AI neuron was consistent in one category under the three tested sound levels, we assigned the azimuth preference of the neuron into that category. For some AI neurons, the azimuth preferences were varied under the three tested sound level conditions, so we classified their azimuth preference as “mixed preference.” Based on this classification scheme, the proportions of AI neurons with different azimuth preference in the four group of rats were shown in Figure 6D. These data demonstrated that most of the AI neurons in the two control groups preferred stimuli from contralateral azimuth. In contrast, UCHL at young age decreased the proportion of neurons with contra-field preference but largely increased the proportion of neurons with ipsi-field preference in YUCHL group; UCHL at adult age also decreased proportion of neurons with contra-field preference, but largely increased the proportion of neurons with mixed preference in the AUCHL group (Figure 6D).

### The Effect of UCHL at Different Postnatal Ages on the Azimuth Selectivity of AI Neurons

**FIGURE 7:**
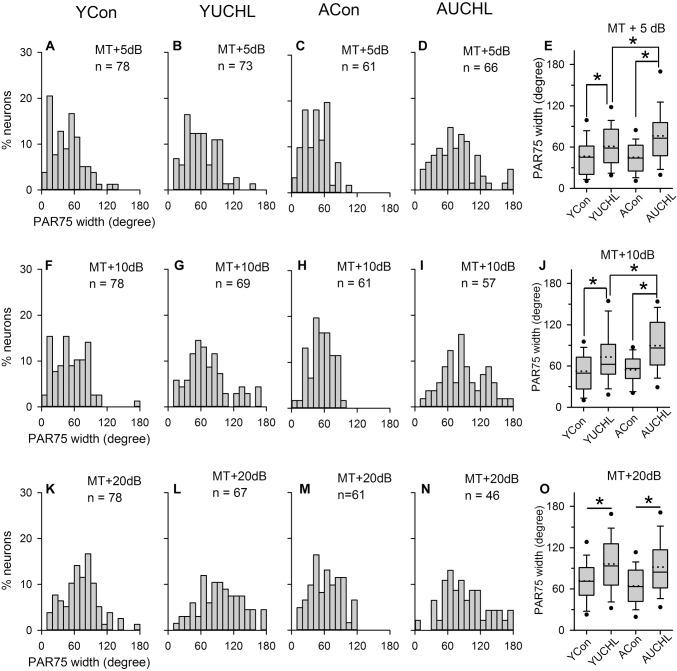
Comparison of the PAR75 width among population of AI neuron from the four groups of rats. The data shown in the panels of the top, middle, and bottom rows are determined at MT+5 dB **(A–D)**, MT+10 dB **(F–I)**, and MT+20 dB **(K–N)** conditions, respectively. Bar histograms show the distributions of PAR75 width of the azimuth functions of A1 neurons from the YCon group (the first column), the YUCHL group (the second column), the ACon group (the third column), and the AUCHL group (the fourth column). **(E,J,O)** The fifth column box plots of the PAR75 width of A1 neurons in the four groups of rats determined at different sound levels. Each box plot shows the median (solid line in the boxes), mean (dotted line in the boxes), quartiles (box extremities), 10th/90th percentiles (error bars), 5th/95th percentiles (filled circles). ^∗^ indicates significant difference between the two groups (Mann–Whitney test, *p* < 0.01).

The PAR75 width is an index to evaluate the azimuth selectivity of AI neurons. The greater the PAR75 width, the weaker the azimuth selectivity. The distributions of the PAR75 width determined from the four groups of AI neurons are shown in Figure 7. The data shown in bar histograms indicated that, under MT+5 dB and MT+10 dB conditions, the PAR75 widths for the majority of the neurons in both the YCon group and the ACon group were ≤60°. In contrast, UCHL decreased the proportion of AI neurons with PAR75 width ≤60° in both the YUCHL group and the AUCHL group. The proportions of neurons with PAR75 width ≤60° in the four groups were as follows: at MT+5 dB condition, YCon vs. YUCHL, 70.51% vs. 53.43%, ACon vs. AUCHL, 70.49% vs. 34.85%; at MT+10 dB condition, YCon vs. YUCHL, 58.97% vs. 40.58%, ACon vs. AUCHL, 59.02% vs. 22.81%; at MT+20 dB condition, YCon vs. YUCHL, 33.33% vs. 16.42%, ACon vs. AUCHL, 45.90% vs. 21.74%.

We performed statistical analysis on the PAR75 width of AI neurons in the four groups by Mann–Whitney test (Figure 7, panels in the fifth column). The results indicated that no significant differences in the PAR75 width were found between the two control groups (YCon vs. ACon: at MT+5 dB, *Z* = -0.121, *p* = 0.904; at MT+10 dB, *Z* = -0.707, *p* = 0.480; at MT+20 dB, *Z* = -1.244, *p* = 0.214). In comparison with the PAR75 width of AI neurons in the YCon group, UCHL at young age significantly increased the PAR75 width of AI neurons in the YUCHL group (YCon vs. YUCHL, at MT+5 dB, *Z* = -3.02, *p* = 0.003; at MT+10 dB, *Z* = -3.276, *p* = 0.001; at MT+20 dB, *Z* = -3.916, *p* < 0.001). Similarly, in comparison with the PAR75 width of AI neurons in the ACon group, UCHL in adulthood significantly increased the PAR75 width of AI neurons in the AUCHL group (ACon vs. AUCHL: at MT+5 dB, *Z* = -4.949, *p* < 0.001; at MT+10 dB, *Z* = -5.270, *p* < 0.001; at MT+20 dB, *Z* = -3.565, *p* < 0.001). Moreover, the effect of UCHL on increasing the PAR75 width was greater in the AUCHL group than in the YUCHL group under both MT+5 dB and MT+10 dB conditions (YUCHL vs. AUCHL: at MT+5 dB, *Z* = -2.375, *p* < 0.001; at MT+10 dB, *Z* = -2.605, *p* < 0.009). However, we did not find significant differences in the PAR75 width of AI neurons between the YUCHL group and the AUCHL group under MT+20 dB condition (YUCHL vs. AUCHL: *Z* = -0.777, *p* = 0.43).

### The Effect of UCHL at Different Postnatal Ages on the Azimuth Sensitivity of AI Neurons

**FIGURE 8:**
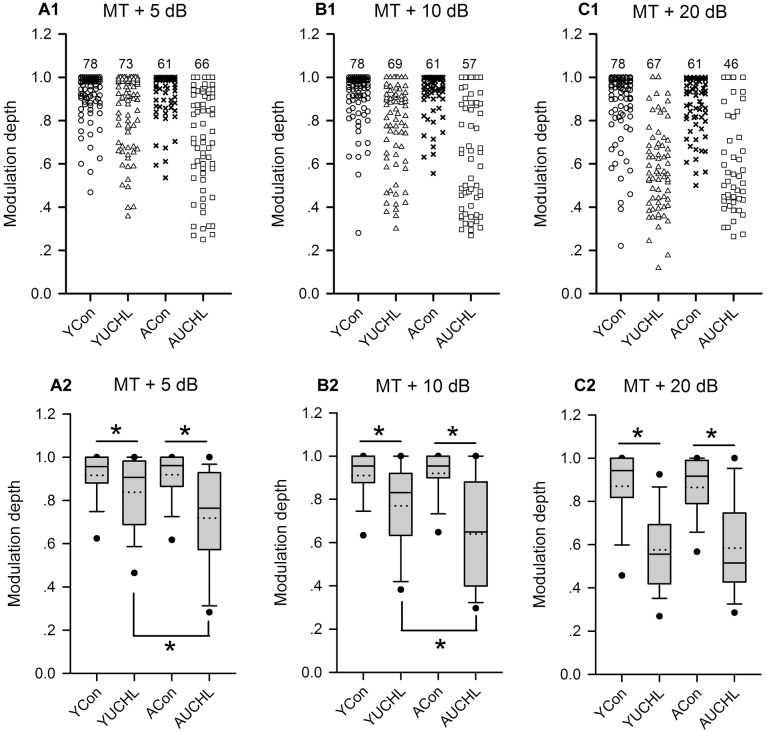
Comparison of the modulation depth of the azimuth functions of AI neurons among the four groups of rats. For each panel, the *x*-axis indicates four different groups of rats, and the *y*-axis represents the value of the modulation depth. MT+5 dB, MT+10 dB, and MT+20 dB are the sound levels used to determine the azimuth functions of AI neurons. **(A1–C1)** The data of modulation depth for each group determined at the tested sound levels, and the number on the top of the symbols for each group is the number of neurons included. **(A2–C2)** The box plots showing the comparison of the modulation depth for the population AI neurons in the four groups of rats. The plots show the median (solid line in each box), mean (dotted line in each box), quartiles (box extremities), 10th/90th percentiles (error bars), and 5th/95th percentiles (filled circles). ^∗^ indicates significant difference in modulation depth between the two groups (Mann–Whitney test, *p* < 0.05).

The modulation depth of the azimuth function indicates the azimuth sensitivity of an AI neuron. The higher the modulation depth, the greater the sensitivity to sound source azimuth. The data of the modulation depth from the four groups of AI neurons indicate that the ranges of modulation depth were larger in the two groups with UCHL than in the two control groups (Figure 8A1–C1). The proportions of AI neurons with high modulation depth (arbitrary ranges: 0.9–1.0) were as follows: at MT+5 dB, YCon vs. YUCHL, 70.51% vs. 53.43%, ACon vs. AUCHL, 65.57% vs. 31.82%; at MT+10 dB, YCon vs. YUCHL, 69.23% vs. 34.78%, ACon vs. AUCHL, 77.05% vs. 21.05%; at MT+20 dB, YCon vs. YUCHL, 62.82% vs. 7.46%, ACon vs. AUCHL, 50.82% vs. 15.22%. The data indicated that the modulation depth for most of the AI neuron in the two control groups was distributed within the range of 0.9–1.0. In contrast, UCHL decreased the proportion of AI neurons with high modulation depth in both the YUCHL group and the AUCHL group.

The statistical comparison for the modulation depth among the four groups of AI neurons were performed by box plots and by Mann–Whitney test (Figure 8). We found no significant differences in modulation depth between the two control groups (YCon vs. ACon: at MT+5 dB, *Z* = -0.201, *p* = 0.841; at MT + 10 dB, *Z* = -0.250, *p* = 0.803; at MT+20 dB, *Z* = -0.893, *p* = 0.372). In comparison with the modulation depth in the YCon group, UCHL significantly decreased the modulation depth of AI neurons in the YUCHL group (YCon vs. YUCHL: at MT+5 dB, *Z* = -3.306, *p* = 0.002; at MT+10 dB, *Z* = -5.177, *p* < 0.001, MT+20 dB, *Z* = 7.665, *p* < 0.001). Similarly, in comparison with the modulation depth in the ACon group, UCHL significantly decreased the modulation depth of AI neurons in the AUCHL group (ACon vs. AUCHL: at MT+5 dB, *Z* = -5.742, *p* < 0.001; at MT+10 dB, *Z* = -6.108, *p* < 0.001; at MT+20 dB, *Z* = -5.954, *p* < 0.001). Moreover, under the conditions of both MT+5 dB and MT+10 dB, the modulation depth of AI neurons was greater in YUCHL group than in AUCHL group (YUCHL vs. AUCHL: at MT+5 dB, *Z* = -3.353, *p* = 0.001; at MT+10 dB, *Z* = -2.830, *p* = 0.005). However, under MT+20 dB condition, we did not find significant differences in the modulation depth between the YUCHL group and the AUCHL group (*Z* = -0.207, *p* = 0.836). These data demonstrated that UCHL decreased the azimuth sensitivity of AI neurons, and this effect was greater in the AUCHL group than in the YUCHL group.

## Discussion

Conductive hearing loss produces a partial attenuation of acoustic input to the affected ear (Moore et al., 1989), and UCHL is quite common in human infants with otitis media (Gravel and Wallace, 2000). In animal studies, the disruption of the normal acoustic conduction in the middle-ear has been a common manipulation of conductive hearing loss. The effects of UCHL on the structure and function of the auditory system have been evaluated in the cochlea (Liberman et al., 2015), as well as in the brainstem and auditory cortex (Moore et al., 1989; Tucci et al., 1999; Hutson et al., 2008; Popescu and Polley, 2010; Polley et al., 2013; Yao and Sanes, 2018).

In the present study, we determined the azimuth functions of rat AI neurons and assessed the neural tuning to sound source azimuth by the azimuth preference, the PAR75 width, and the tuning depth of the azimuth functions. We did not find significant differences in the azimuth tuning of AI neurons evaluated by these measurements between the two control groups. The results implied that the age difference between the two control groups did not induce significant changes in the azimuth tuning of rat AI neurons. In comparison with the data in age-matched control groups, the data in both YUCHL group and AUCHL group showed that chronic UCHL induced a lower proportion of contra-field preference neurons in AI, and increased the PAR75 width but decreased the tuning depth of the azimuth functions of AI neurons contralateral to the operated ear. Moreover, UCHL induced a larger percentage of ipsi-field preference neurons in the AI contralateral to the operated ear in the YUCHL group than in the AUCHL group. These results demonstrated that chronic UCHL significantly disrupted the azimuth tuning of AI neurons contralateral to the operated ear, and the effects of UCHL on the azimuth tuning of AI neurons was age-related. The different effects of UCHL on the azimuth tuning of AI neurons shown in developing rats and in adult rats indicated that the timing of UCHL played a critical role in mediating its effects. To our knowledge, the present study is the first demonstration of how chronic UCHL at different postnatal ages affects the azimuth tuning of AI neurons in free-field acoustic stimulus conditions.

In the present study, the responses of AI neurons to stimuli from the azimuths ipsilateral to the operated ear in the average azimuth functions were greater in the two UCHL groups than in the two control groups (Figure 5). Interestingly, the responses to stimuli from ipsilateral azimuths were greater in the YUCHL group than in the AUCHL group. Consistent with these changes found in the auditory cortex of UCHL rats, previous studies in the inferior colliculus of ferrets showed that unilateral cochlear removal in adults ferrets led to a rapid and dramatic increase in the proportion of neurons ipsilateral to the intact ear, and unilateral cochlear removal in infancy led to a further increase in responsiveness of individual neurons ipsilateral to the intact ear (McAlpine et al., 1997). We speculate that the changes in responses induced by UCHL in the present study were probably due to both a decrease of inhibition to the ipsilateral input and an increase of ipsilateral input. Because UCHL or unilateral deafness at young age induced an aural preference to the ipsilateral normal hearing ear (Popescu and Polley, 2010; Kral et al., 2013b; Tillein et al., 2016), the greater responses in the YUCHL group than in the AUCHL group at each of the ipsilateral azimuths (Figure 5) were possibly due to a greater increase of ipsilateral input in the YUCHL group than in the AUCHL group.

Previous studies in cats related to the present study demonstrated that congenital monaural and binaural deafness resulted in a decrease in sensitivity of AI neurons to interaural time differences (Tillein et al., 2010, 2016). This reduced sensitivity in the AI contralateral to the deaf ear was more pronounced in congenitally monaural deaf cats than in congenitally binaural deaf cats (Tillein et al., 2016). Therefore, both UCHL and unilateral deafness at young age exert significant impacts on the spatial tuning of AI neurons in adulthood.

### Neural Tuning to Sound-Source Azimuth in the Auditory Cortex of Normal Hearing Animals

In the present study, the azimuth functions of AI neurons obtained from two control groups demonstrated that more than half of the neurons in the rat AI preferred sound stimuli from the contralateral azimuth in the horizontal plane. This is consistent with the findings shown in previous studies regarding the spatial representation of rat AI neurons measured by spike counts (Yao et al., 2013; Gao et al., 2018) and excitatory postsynaptic potentials (Chadderton et al., 2009). Studies using interaural level differences to mimic the changes of spatial azimuth also demonstrated that most tested neurons in the rat AI preferred acoustic stimuli from contralateral horizontal positions (Kelly and Sally, 1988). Although the criteria used to define the azimuth preference were varied among these studies, the results reached the similar conclusion that most neurons in the rat AI favored the acoustic stimuli in contralateral frontal auditory space. Studies in other species such as cats showed that most neurons in the AI of anesthetized cats responded best to sound stimuli from contralateral azimuths, and a small proportion of neurons preferred sound stimuli from the midline or ipsilateral azimuths (Imig et al., 1990; Rajan et al., 1990; Clarey et al., 1995; Eggermont and Mossop, 1998). These findings obtained in free-field studies were also demonstrated from close-field studies in anesthetized cats by manipulating the interaural level differences (Zhang et al., 2004). Moreover, the overrepresentation to the contralateral field in horizontal plane in AI was further shown in awake cats (Mickey and Middlebrooks, 2003) and alert macaque monkeys (Woods et al., 2006).

Taken together, the overrepresentation of contralateral azimuth is a common feature of neural spatial tuning in AI. This feature has been demonstrated in different animal species, and in both awake and anesthetized conditions. Therefore, any manipulations that induce a change of this feature would imply its consequences of abnormal auditory spatial tuning in AI.

### The Effect of UCHL at Young Age on the Azimuth Tuning of AI Neurons

In contrast to the common feature of the overrepresentation of contralateral azimuth in the AI of normal hearing rats, here we show that chronic UCHL in P14 rats induced a significant decrease in the proportion of contra-field preference neurons and a significant increase in the proportion of ipsi-field preference neurons in the AI contralateral to the operated ear (Figure 6). The mean normalized azimuth functions for the population AI neurons in the YUCHL group showed a clear preference for the stimuli from the ipsilateral azimuths under MT+5 dB and MT+10 dB conditions (Figure 5). These changes in azimuth functions in YUCHL group implied an augment of the responsiveness of AI neurons to acoustic input from the ipsilateral non-operated ear due to UCHL at early postnatal age. The results also showed that the PAR75 width for the azimuth functions of AI neurons was significantly greater in the YUCHL group than in the age-matched YCon group (Figure 7), indicating a down regulation of azimuth selectivity by UCHL at early postnatal age. Moreover, UCHL induced a reduction in modulation depth of azimuth functions in the YUCHL group (Figure 8), indicating a degraded sensitivity to the change of sound source azimuth. Taken together, UCHL at early postnatal age disrupts the normal spatial tuning of AI neurons to sound source azimuth in adulthood. Related studies in deaf cats showed that both monaural and binaural congenital deafness decreased the sensitivity to interaural time differences in the auditory cortex. The modulation depth in the functions of interaural time differences in the auditory cortex was smaller in congenitally monaural and binaural deaf cats than in hearing control cats (Tillein et al., 2010, 2016). Thus, it is quite clear that postnatal abnormal binaural input results in degraded representations of sound source location in the auditory cortex.

Previous closed-field studies in rodents have demonstrated an experience-dependent plasticity in frequency tuning in AI during postnatal hearing development (Popescu and Polley, 2010; Polley et al., 2013). In normal binaural hearing rats, the AI displayed poor-organized tonotopic maps and low-quality frequency receptive fields in responding to tonal stimuli from the ipsilateral ear. However, chronic UCHL by ear canal ligation in P14 or P28 rats weakened the representation of the contralateral ligated ear in AI and strengthened the representation of the ipsilateral open ear in AI. This was exhibited in adulthood by well-organized ipsilateral tonotopic maps and good quality ipsilateral tonal receptive fields in the AI contralateral to the ligated ear (Popescu and Polley, 2010). Further studies showed that short-term reversible UCHL during the critical periods of mouse hearing development was associated with enhanced sensitivity of AI neurons to the ipsilateral non-operated ear in adulthood (Polley et al., 2013). Studies in cats with unilateral deafness also showed that a massive reorganization of aural preference in favor of the hearing ear was found if the onset of unilateral hearing was before or around the peak of functional synaptogenesis (Kral et al., 2013b), and that unilateral hearing experience led to a functionally asymmetric AI with different neuronal reorganizations and different sensitive periods involved (Kral et al., 2013a). These studies in rodents and cats demonstrated a developmental experience-dependent plasticity in AI from the perspective of frequency tuning and binaural selectivity. The results in the present study provide new evidences for the developmental experience-dependent plasticity in AI from the perspective of azimuth tuning.

At present, we have no direct evidences on the mechanism underlying the effects of chronic UCHL at young age on the azimuth tuning of rat AI neurons. Previous studies showed that chronic UCHL in neonatal ferrets and rhesus monkey did not significantly change the neuronal size of brainstem auditory neurons or the volume of the cochlear nuclei (Moore et al., 1989; Doyle and Webster, 1991). Whereas UCHL in neonatal ferrets in the right ear did not lead to degeneration of the cochlear nucleus on the right side, it led to a significant rearrangement of brainstem connectivity, i.e., a significant increase of projections in the left inferior colliculus from left cochlear nucleus (Moore et al., 1989). Even transient UCHL in developing animals could induce dramatic changes in the structure and function of auditory system. For example, cochlear nucleus neurons innervated by auditory nerve redistributed synaptic AMPA and glycine receptor in response to transient UCHL in P30 rats induced by earplugging (Whiting et al., 2009). Short-term monaural earplugging in P30 rats altered the synthesis and subsequent composition of specific glutamate and glycine receptors and the synapses in the ipsilateral cochlear nucleus (Wang et al., 2011), and led to long-lasting structure and molecular alterations at the endbulb of the Held synapse (Clarkson et al., 2016). In addition, the short-term earplugging in P30 rats induced an increase in postsynaptic density thickness and the upregulation of the postsynaptic GluA3 AMPAR subunit (Clarkson et al., 2016). Early developmental UCHL also resulted in a weakening of neurophysiological representation in the auditory system corresponding to the affected ear. Studies using 2-deoxyglucose uptake as a measure of metabolic activity have shown that, 3 weeks of UCHL induced by malleus removal in P21 gerbils resulted in a marked decrease in glucose uptake in the major ascending projections in the brainstem of the operated ear (Tucci et al., 1999), and in both the inferior colliculus and auditory cortex contralateral to the operated ear (Hutson et al., 2008). These results suggest that neural circuits responsible for integrating input from the two ears may be particularly susceptible early in development, and the abnormal binaural input during early postnatal age could induce plasticity in both structural and functional changes in the central auditory system. In the present study, we found dramatic changes in azimuth tuning of AI neurons induced by UCHL in developing rats, we postulate that these changes in azimuth tuning might be caused by the developmental experience-dependent plasticity in the structure and function of the central auditory system beginning from the cochlear nucleus all the way to the auditory cortex.

### The Effect of UCHL in Adulthood on the Azimuth Tuning of AI Neurons

Compared to the data in ACon group, the data in AUCHL group indicated that chronic UCHL in adult rats induced a complex effect on azimuth tuning of AI neurons contralateral to the operated ear: (1) a reduction in the proportion of contra-field preference neurons and an increase in the proportion of mixed preference neurons; (2) an increase in the proportion of ipsi-field preference neurons; (3) an increase in the PAR75 width and a decrease in the modulation depth of azimuth functions. Interestingly, chronic UCHL in adult rats induced a larger increase in the percentage of mixed preference neurons than chronic UCHL in young rats did. In contrast, chronic UCHL in young rats induced a larger increase in the percentage of ipsi-field preference neurons than chronic UCHL in adult rats did. These results suggest that UCHL in developing rats results in a greater weakening of neurophysiological representation associated with the affected ear. These age-dependent effects of UCHL on the azimuth tuning of AI neurons was probably due to the differences in the UCHL-induced plasticity of central auditory system of rats between the YUCHL group and the AUCHL group.

Unilateral conductive hearing loss attenuates the input to the inner ear and alters the normal binaural integration in the central auditory system. Previous studies in adult cats showed that many AI neurons that were sensitive to sound source azimuth in normal binaural hearing conditions immediately became insensitive to azimuth (i.e., either responded well to all azimuths or responded poorly at any azimuth) when one ear was plugged (Samson et al., 1994). Thus, the acute UCHL by earplugging disrupted the normal binaural interaction, and immediately changed the azimuth tuning of cat AI neurons. In contrast to acute UCHL that operated for a few minutes, chronic UCHL that lasts for weeks or months could induce plasticity in the adult central auditory system due to the attenuation of auditory input. Previous studies have shown that, in the fully developed ears of adult mice, the chronic UCHL induced a significant plasticity of the cochlea’s afferent and efferent innervations. Chronic UCHL induced by removal of tympanic membrane increased the cochlear-nerve synaptopathy in the ipsilateral ear, and reduced the density of olivocochlear bundle (Liberman et al., 2015). Moreover, chronic UCHL in adult gerbils by malleus removal caused a decrease in metabolic activity measured by 2-deoxyglucose uptake in the cochlear nucleus, lateral lemniscus, and inferior colliculus that primarily receive excitatory inputs from the operated ear (Tucci et al., 1999). UCHL in adult rats also caused a shift in aural dominance in the auditory cortex (but not in the inferior colliculus) contralateral to the operated ear (Popescu and Polley, 2010). These studies demonstrated that chronic UCHL induced a significant plasticity in the adult central auditory system. In the present study, the chronic UCHL in adult rats induced more complex effects on azimuth tuning of AI neurons than the acute UCHL in adult cats did, which suggests that the spatial hearing remains plastic in adulthood. We speculate that the effects of chronic UCHL in adult rats on the azimuth tuning of AI neurons may be attributed to the consequences of UCHL on both the abnormal binaural acoustic input and the experience-dependent plasticity in the adult central auditory system. In the present study, we did not investigate the impacts of acute UCHL on the azimuth tuning of rat AI neurons. Therefore, whether the effects of chronic UCHL in adult rats on the azimuth tuning of AI neurons were indeed caused by the plasticity in AI needs to be further investigated.

### Methodology Considerations

In the present study, we focused on investigating the effects of UCHL on the azimuth tuning of AI neurons contralateral to the operated ear because the excitatory drive from the contralateral ear is predominant in the auditory cortex in normal binaural hearing conditions. Previous studies in ferrets showed that unilateral cochlear removal in infancy led to an increase in responsiveness of individual neurons in the ipsilateral inferior colliculus, but had no significant impacts on the responses in the inferior colliculus contralateral to the intact ear (McAlpine et al., 1997). In unilateral deaf cats, the cortex ipsilateral to the hearing ear showed responses to stimulation from both the hearing as well as the deaf ear (Kral et al., 2013b) whereas the cortex contralateral to the hearing ear showed good responsiveness to the hearing ear but weak responses to stimulation of the deaf ear (Kral et al., 2013a). Moreover, congenital unilateral deafness in cats induced a stronger reduction in the sensitivity to interaural time differences in the auditory cortex ipsilateral to hearing ear than in the auditory cortex contralateral to the hearing ear (Tillein et al., 2016). These studies demonstrated a hemispheric specificity of the impacts of single-sided deafness. In the present study, we did not measure the azimuth functions of AI neurons ipsilateral to the operated ear. It’s possible that the UCHL also affects the azimuth tuning of AI neurons ipsilateral to the operated ear (or contralateral to the intact ear) due to the attenuation of inhibitory drive or excitatory drive from the operated ear. The AI neurons ipsilateral to the operated ear receive excitatory drive predominantly from the contralateral intact ear, therefore we postulate that the impacts of UCHL on the azimuth tuning of the AI neurons ipsilateral to the operated ear could be weaker than the impacts shown in the present study.

In the single-sided congenital deaf cats, sensitivity of AI neurons to the interaural time differences was reduced, particularly at the AI ipsilateral to the hearing ear (Tillein et al., 2016). In the present study, we used rats as the experimental animals and the CFs of the rat AI neurons included in the data analysis were ≧4 kHz. Therefore, the impacts of UCHL on the azimuth functions of the rat AI neurons primarily reflected the impacts on the sensitivity and selectivity of interaural level differences, and the impacts of UCHL on the sensitivity of the interaural time differences of AI neurons are not known in the present study.

In the present study, the number of neurons in each group were within the range of 61–78. Due to the limited time that a well-isolated neuron was available for recording in this acute experiment, we only got few neurons for data analysis in each rat. If more neurons were recorded in each rat, the power for the statistical analysis would be higher than the present situation.

## Conclusion

In summary, the present study demonstrated that postnatal chronic UCHL at both young age and adult age disrupts the normal neuronal tuning to sound source azimuth in the AI contralateral to the operated ear. Chronic UCHL altered the azimuth preference, degraded the azimuth sensitivity and azimuth selectivity of AI neurons. In addition, UCHL at early postnatal age induced a greater weakening of cortical neurophysiological representation associated with the affected ear than UCHL in adulthood did, indicating age-related effects of UCHL on cortical spatial tuning. The disruption of the azimuth tuning of AI neurons induced by chronic UCHL may be one of the neural mechanisms underlying the poor sound localization abilities shown in chronic UCHL subjects.

## Ethics Statement

All procedures used in the present study were approved by the Institutional Animal Care and Use Committee of East China Normal University, and were in accordance with the National Institute of Health Guide for the Care and Use of Laboratory Animals (NIH Publications No. 80-23) revised 1996. All efforts were made to minimize the suffering of animals and the number of animals used.

## Author Contributions

JZ and XW designed this study, analyzed the data, and drafted the manuscript. XW and JL engaged in data collection. All authors contributed to the final version of the manuscript and approved its contents.

## Conflict of Interest Statement

The authors declare that the research was conducted in the absence of any commercial or financial relationships that could be construed as a potential conflict of interest.

## Abbreviations

ABR: auditory brainstem response
ACon: adult control
AI: primary auditory cortex
AUCHL: adult unilateral conductive hearing loss
CF: characteristic frequency
MT: minimum threshold
PAR75: the preferred azimuth range over which the normalized response strengths are ≥0.75
UCHL: unilateral conductive hearing loss
YCon: young control
YUCHL: young unilateral conductive hearing loss
